# Cognitive Reserve and Psychological Well‐Being in Aging Population of Community‐Dwellers in Spain

**DOI:** 10.1002/brb3.71714

**Published:** 2026-09-02

**Authors:** Mikhail Ordin, Angel Barrasa, Caridad López‐Granero, Leona Polyanskaya

**Affiliations:** ^1^ Faculdade de Medicina Universidade de Coimbra Coimbra Portugal; ^2^ Interdisciplinary Centre for European Sudies Europa Universität Flensburg Deutschland; ^3^ UTAMED Universidad Tecnológica Atlántico‐Mediterráneo, Málaga Spain; ^4^ Department of Psychology and Sociology Universidad de Zaragoza Teruel Spain; ^5^ Department of English and German Studies and Translation & Interpreting University of the Basque Country UPV/EHU Vitoria‐Gasteiz Spain

**Keywords:** anxiety, cognitive decline, cognitive reserve, healthy aging, life satisfaction, loneliness

## Abstract

**Introduction:**

Greater brain resilience—the capacity of the brain to maintain normal functioning and cognition with aging—is associated with a slower cognitive decline. Cognitive reserve (CR) is one component of brain resilience and may be supported by educational, occupational, and leisure experiences.

**Purpose:**

This exploratory, cross‐sectional study examined associations among psychological well‐being, engagement in leisure activities associated with cognitive reserve, and cognitive performance in older community dwellers (older adults living independently) in Spain. General brain resilience was tapped on by measuring performance in memory and attention cognitive tasks.

**Method:**

We used the depression, anxiety and stress scale (DASS‐21), life satisfaction scale (SWLS), loneliness scale (UCLA), and cognitive reserve scale (CRS) as proxy measures for psychological well‐being and cognitive reserve in 80 people (33 men) between 65 and 101 years of age. The CRS was used as a proxy measure of the leisure‐activity contribution to cognitive reserve rather than as a comprehensive or direct measure of cognitive reserve. To probe cognitive performance, we administered a memory‐span task and spatial‐cueing tasks measuring endogenous and exogenous attention.

**Findings:**

Among males, higher CRS scores were associated with lower perceived stress and greater reported loneliness, whereas comparable associations were not observed among females. Higher CRS scores were also associated with selected measures of attention. No significant association was observed between CRS scores and memory span. Given the exploratory design and sample size, sex‐specific findings should be interpreted cautiously.

**Conclusion:**

We discuss these findings and outline a research agenda for more hypotheses‐driven studies to investigate the intricate relationships among different aspects of brain resilience, psychological well‐being and cognitive performance in older individuals. Better understanding of intricate associations between cognitive performance and psychological factors (feeling of loneliness, subjective stress and anxiety, and life satisfaction) may contribute to our understanding of how lifestyle and cognitive reserve are related.

## Graphical abstract

Psychological well‐being is associated with higher engagement into physical and cognitive activities in males than in females. We observed association between engagement into such activities and attention, but not memory.

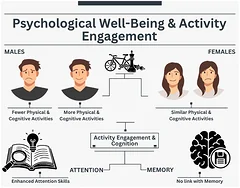

## 1 Introduction

Aging is often associated with a decline in cognitive functions, which, however, develops at different rates in different individuals. The ability of the brain to maintain cognitive functions with age is referred to as brain resilience (Stern et al. 2023), which is often attributed to three relatively independent concepts:

- Cognitive reserve (the ability of the brain to perform cognitive tasks despite neurodegenerative changes, disease or injury, i.e., the ability to maintain cognitive functioning when the anatomical structures are damaged or impaired). Cognitive reserve is a broad construct that is commonly estimated using indicators such as educational attainment, occupational complexity, and participation in cognitively, socially, and physically stimulating leisure activities;
- Brain maintenance (the ability of the brain to withstand age‐related neurodegenerative processes and resist associated anatomical changes, i.e., the ability of the brain to maintain anatomical integrity in aging and preserve cognitive functions); and
- Brain reserve (the strength of structural connections and the number of active neurons and synapses, which can be recruited for cognitive functions).

Brain resilience—across all three components—is modulated by genetic, environmental, psychological, and lifestyle factors. We will now consider these concepts related to brain resilience in more detail.

Brain morphology (Peters 2006; Sivera et al. 2019; Zhao et al. 2018), connectivity patterns (Addis et al. 2010; Edde et al. 2021), and even lateralization (Szaflarski et al. 2006) change with age, which may result in behavioral and cognitive changes. Frequently, such changes are negative, with older people exhibiting varying degrees of severity of cognitive decline, and age‐related cognitive impairments are often associated with increased impulsivity, disinhibition, and emotional instability (Sakurai et al. 2020; Seaman et al. 2022). However, some individuals are able to withstand the negative effects of age‐related changes in the central nervous system better than others are able to. In a pivotal study, Katzman et al. (1988) reported that morphological and histological properties in postmortem brains, which are associated with Alzheimer's disease, were not associated with abnormal behavior or cognitive problems in individuals when they were alive. This finding suggested that in some individuals, anatomical and physiological degenerative changes at the neural level may not exhibit phenotypic expression. Later studies revealed that individual differences in regaining functionality after brain surgery or stroke affect the same neural substrates (Bigio et al. 2002). Cognitive reserve, or the ability of the brain to resist age‐ or disease‐related neurological damage and maintain normal cognitive performance (Staff et al. 2004; Opdebeeck et al. 2015), can be increased via plasticity, the development of differential processing strategies or the recruitment of differential neural networks to perform the same cognitive task (Barulli and Stern 2013).

In other words, cognitive reserve refers to the brain's normal functioning despite the presence of neurodegenerative or traumatic changes. However, the brain can also withstand such neurodegenerative changes in morphology, and the brain with an unaffected morphology is more likely to maintain normal cognition in healthy aging. This is often referred to as brain maintenance (Gazes et al. 2023; Habeck et al. 2017). Brain maintenance is determined by lifestyle choices to a greater degree than by cognitive reserve, although both can be improved by regularly practicing certain mental and physical activities that are associated with better mental health and slower neurodegenerative decline (Scarmeas et al. 2003; Wilson et al. 2003). Cognitive reserve is often operationalized through several complementary indicators, including education, occupational attainment or complexity, and participation in cognitively stimulating leisure activities. The present study focuses specifically on the leisure‐activity component. The extent to which individuals have participated in such activities across their lifetime was assessed using the questionnaire developed by León et al. (2014) and was treated as a proxy for the leisure‐activity contribution to cognitive reserve. Thus, the measure used here should not be interpreted as a comprehensive assessment of cognitive reserve, as it does not incorporate education‐ or occupation‐related indicators.

Another concept that is frequently recurring in the literature is the brain reserve, or the ability of the brain to withstand neurodegenerative damage before morphological changes start affecting cognition (Hachinski and Avan 2022; Stern et al. 2020). The brain reserve is related to the cognitive reserve as hardware and software, respectively: more powerful hardware will still be functional if the script is badly written, but an optimized script can be efficient even if the hardware is less powerful. Maintenance is the ability of hardware not to be broken by physical damage, due to better materials, bigger size, or protection measures. Taken together, they provide a life‐long accumulating capacity to resist physical, emotional, and psychological harm in a dynamic and interactive manner, or brain resilience.

A decline in cognitive function can be caused by factors that are not related to neurodegenerative changes or mediated by a variety of environmental (Calderón‐Garcidueñas et al. 2008; Guxens et al. 2018; Mortamais et al. 2019; Peterson et al. 2015; Yao and Hsieh 2019) and ontogenetic (Rice and Barone 2000; Grandjean and Landrigan 2014; Dekker and Karmiloff‐Smith 2010; Power et al. 2016; Dillon et al. 2014) influences. In addition, neurodegeneration can be accelerated by constant psychological distress (Hsieh et al. 2024; Power et al. 2015; Scott et al. 2015; Sussams et al. 2020; Yao et al. 2024). Education‐related factors (Stern et al. 1992) and lifestyle and the residential environment (Craik et al. 2010; Hsieh et al. 2021; Hsieh et al. 2024; Scarmeas and Stern 2003) can help individuals withstand degenerative processes. Although education and occupational activity are important contributors to cognitive reserve, the present study does not examine these dimensions and is restricted to leisure activities that may contribute to cognitive reserve. Sex and gender have also been reported to influence cognitive reserve (Chen et al. 2017; Wang et al. 2020), with females resisting degenerative changes more efficiently than men do. There is still no consensus on whether these factors are social (e.g., social roles assigned to and played by people, with some activities or behavioral patterns being more typical of males or females), biological (e.g., anatomical differences between males and females), or both. Furthermore, we refer to sex, but the choice of the term is made under uncertainty as to whether these factors are social or biological. For a fuller overview of the factors that support cognitive reserve, see Staff et al. (2004) and Hsieh et al. (2024).

In this study, we aimed to examine how psychological and lifestyle factors are associated with participation in leisure activities that may contribute to cognitive reserve. The study therefore addresses a specific leisure‐activity proxy of cognitive reserve rather than the full construct, which may also encompass educational and occupational experiences. In particular, we set out to explore (1) how a variety of psychological and lifestyle factors are associated with participation in mental, social, and physical leisure activities that may help protect against age‐related cognitive decline (León et al. 2014), and (2) how participation in these activities is associated with older adults’ performance in cognitive tasks.

## 2 Materials and Methods

### 2.1 Participants

**FIGURE 1:**
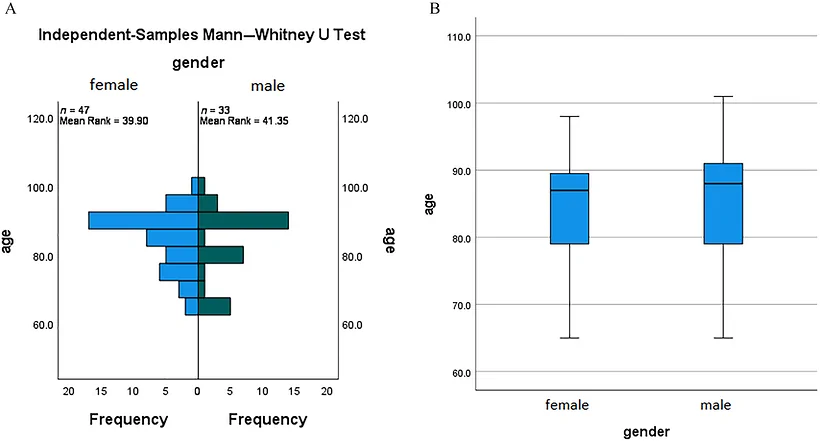
Age distribution (a), showing how many participants fall into each age data bin for each sex; and summary data for each sex (b), with whiskers showing lower and upper 25% of data, and horizontal line showing the median age for each group.

The sample under study consists of 80 Spanish elderly people (33 men). The participants lived independently, and none of them resided in assisted living facilities. A total of 73.3% shared their household with family members (64% were married, others were either widowed or divorced/separated and lived with other family members), and 26.7% of the participants lived alone. Among all participants (living solitary and in shared households), 20% were widowed, 10.7% were single, and 5.3% were separated or divorced. With respect to the educational level of the sample, 40% of the elderly people had primary studies, 22.7% had secondary studies, 18.7% had university studies, and 18.7% had no studies. A total of 13.7% were still working, and the others were retired. Sixty‐five percent of all participants reside in urban areas (*n* = 52), of whom 15 men and 37 women, and 18 men, and 10 women reside in rural areas (i.e., most women are from urban areas, whereas men from urban and rural areas are equally represented in our sample). None of the participants changed their residence in the 10 years prior to the study, and only 5 participants (out of 80) changed their residence from rural to urban or from urban to rural in the course of life. Women were between 65 and 98 years old (Mdn = 87, *M *= 84.5, SD = 8.12, SE = 1.18), and men were between 65 and 101 years old (Mdn = 88, *M *= 84 years, SD = 10.33, SE = 1.8). In both sex groups, the variance was homogeneous (based on the median, *p *= 0.195; on the mean, *p* = 0.082; and on the 5% trimmed mean, *p* = 0.078), according to Levene's tests. The Mann‒Whitney test did not reveal any significant differences in age between males and females, *U* = 803.5, *p *= 0.784 (nonparametric tests were used whenever deviations from normality in the data were detected via Shapiro‒Wilks tests). Figure 1 shows the distribution of age values in both sex groups (a) and a comparison of the means and medians between the groups (b). Most participants went only through the primary educational system; we do not have variability in educational level in the sample.

Participants were community‐dwelling older adults who reported general well‐being and no major diagnosed medical conditions requiring ongoing treatment. This classification was based on self‐report and should not be interpreted as evidence of clinically verified healthy aging. No formal cognitive screening, neurological examination, vascular‐risk assessment, or systematic medication review was conducted.

### 2.2 Measurements: Questionnaires and Cognitive Tests

Recruitment was done via the social center for leisure time. A research assistant who lived in the area spread the information among elderly people who attended the social center, and these people then spread it among their friends and relatives. Those who wanted to participate contacted the researcher directly. The researcher then agreed on the time and date that is convenient for participants. Each participant always performed the tests face‐to‐face with the research assistant. There was always only two people in the room—a participant and the research assistant. The tests / interviews / questionnaires were administered either at the participant's home, or in the social center in a quiet room, in two separate sessions on two different days—one for the questionnaires (CRS, UCLA, and DASS‐21) and interviews, and the other for cognitive tests. The questionnaires were administered on paper. Either the participant filled in the questionnaires him/herself using a pen, or the researcher filled in the questionnaire by eliciting the answers from a participant in an interview. The tests were administered on a computer, the screen was placed at a comfortable distance for each participant, and the answers were registered on a keyboard. None of the participants had impaired mobility/dexterity or impaired vision that was not correctable with normal vision aids (glasses) and prevented them from doing the task. Below, we outline these tests and measurements in detail.

We chose the tests that are brief and suitable for older adults and tap on two core cognitive domains that are known to be most sensitive to aging: memory (we only measure memory span) and attention (endogenous and exogenous). Memory span test probes operational memory in a simple way, while still remaining a sensitive standardized measure of immediate verbal memory and working memory capacity. Age‐related decline has been reported for memory span, which makes it diagnostic of neurodegenerative phenotypes (Hester et al. 2004). To probe attention, we used a Posner cueing paradigm as a well‐established measure of visuospatial attentional orientation. This is particularly relevant in older adults because cognitive aging is associated with changes in processing speed and attentional control (Curran et al. 2001). Together, these tasks allowed us to assess cognitive performance using brief, low‐burden paradigms suitable for an elderly population while targeting cognitive mechanisms theoretically and empirically linked to aging (Park and Reuter‐Lorenz 2009).

#### 2.2.1 Cognitive Reserve Scale (CRS)

Certain activities, when practiced regularly, are associated with a slow neurodegenerative decline and hence are often used as a proxy for the cognitive reserve (Scarmeas et al. 2003; Wilson et al. 2003). León et al. (2014) developed a cognitive reserve scale (CRS) adapted for Spanish‐speaking individuals to assess how much individuals are engaged in such activities, and we used this tool for our study. This scale is not a direct measure of the brain's capacity to tolerate age‐related or disease‐related changes; instead, it is a proxy measure of this capacity, based on engagement in reserve‐related activities, hence providing only an indirect measure of cognitive reserve. The CRS consists of 24 questionnaire items, presenting diverse varieties falling into four categories, such as reading, playing a musical instrument, collecting things, and learning about everything related to the collections, practicing other languages, traveling, and engaging in sport/physical exercise activities. The activities are approximately distributed among four domains: daily life, hobbies, information processing, and socializing. The participants had to indicate how often they were engaged in each of these activities on a 5‐point scale (0, never; 1, at least once a year; 2, at least once a month; 3, at least once a week; 4, three or more times a week; and 5, every day or several times a day) in three different life stages (youth, adulthood, and maturity). Precise cutoff age boundaries for life stages were not imposed (participants had to make their own implicit judgments about age ranges for different stages on the basis of self‐awareness).

At the time of the experiment, all the participants reported that they were in the mature life stage (among the three stages offered). The average score across 24 items represented a cognitive reserve score typical of a particular life stage, and the sum of these three values reflected the total cognitive reserve score. This approach and questionnaire have been validated across several studies (León et al. 2017).

#### 2.2.2 Loneliness Scale (UCLA)

We measured how lonely elderly people feel their lives are (measuring subjective loneliness and perceived social isolation). We used Version 3 of the UCLA loneliness scale (Russell 1996), which is based on 20 questionnaire statements, and with each statement, people have to indicate, on a 4‐point scale (1, never; 2, rarely; 3, sometimes; and 4, always), how often they experience a particular feeling described in the statement. Higher scores indicate a higher degree of perceived loneliness. The total score varies between 20 and 80, with items lower than 40 indicating an uncomfortable degree of loneliness (albeit there are no cutoff points). The UCLA scale was culturally validated for the Spanish population (Sancho et al. 2020).

#### 2.2.3 Depression, Anxiety, and Stress Scale (DASS‐21)

The DASS‐21 is a validated instrument designed for the assessment of long‐term emotional states such as depression, anxiety, and stress (Antúnez and Vinet 2012; Crawford and Henry 2003; Lovibond and Lovibond 1995). It represents a questionnaire with four possible responses on a Likert scale that must be answered according to the extent to which the sentence describes what the person experienced during the last week: (0) it has not happened to me, (1) it has happened to me a little or during part of the time, (2) it has happened to me a lot or during a good part of the time, and (3) it has happened to me a lot or most of the time. The DASS‐21 scale includes three separate subscales and gives three subscores: despression score, anxiety score, and stress score (each varies between 0 and 21). As we were interested in general psychological distress, we used a total DASS‐21 score that was calculated by summing responses across all 21 items (index of general psychological distress). The total score varies between 0 and 63, with higher scores reflecting a higher degree of expression of symptoms related to depression, anxiety, and stress. The DASS‐21 scale has been validated both for research and clinical purposes (Antony et al. 1998; Apóstolo et al. 2006; Clara et al. 2001). The DASS‐21 scale was culturally validated for the Spanish population (Daza et al. 2002; Novy et al. 2001), both for separate subscales and for the general index of psychological distress.

#### 2.2.4 Satisfaction With Life Scale (SWLS)

The SWLS was designed by Diener et al. (1985) to assess an individual's overall satisfaction with life. The scale consists of 5 items that measure subjective well‐being and life satisfaction. For each statement, a participant has to report to what extent (s)he agrees with the statement, from 1 (strongly disagree) to 7 (strongly agree); the final score is the sum of all points, hence it varies between 7 and 35 (both boundaries inclusive). Statements are easy: (1) In most ways my life is close to my ideal; (2) The conditions of my life are excellent; (3) I am satisfied with life; (4) So far, I have gotten the important things I want in life; and (5) If I could live my life over, I would change almost nothing. The questionnaire typically takes 1 min to complete. We used a Spanish adaptation of the scale (Atienza et al. 2000), which demonstrated high test‒retest reliability and internal consistency in research and clinical settings (Buelga et al. Diener et al. 1998; Diener et al. 2018; Martínez and García 2007).

#### 2.2.5 Attention

We administered classical spatial cuing tests within the Posner paradigm (Posner 1980; Golla et al. 2004). We had two versions of the test: probing passive attention and active attention.

In the first version (with exogenous cues), participants look at the middle of the screen and fixate their eyes on the cross. On the left and right, there are squares. The trial starts when one of the squares (on the left or on the right) is highlighted. This automatically attracted the participant's attention to the highlighted square. After a delay (which was either long—350 ms or short—100 ms), a circle appeared either in the highlighted (valid cuing) or in a non‐highlighted (invalid cuing) square. A participant needs to click the right or the left button to indicate whether the circle appeared on the left or on the right.

In this version, the cue is displayed on the periphery of the vision field, and the change in the visual field attracts attention to either the left or right side, which can occur without conscious efforts on the part of the participant to switch attention. This test probes passive attention, which is grabbed by the highlighting of the square and must be disengaged to quickly detect the correct position of the stimulus (the cue is valid in 50% of the trials, which means that the reliability of the cue is at the chance level, and it cannot be used; hence, the best strategy is to disengage attention that is automatically attracted to the cue in the periphery vision field).

The second variant of the test probes active attention. The participants look at the cross in the middle of the screen; then, the cross is changed so that the arrow points either to the right or to the left. The stimulus (circle) then appears either on the left or on the right, and the participant needs to click the left or the right button to indicate where the stimulus appeared. The arrow either cues the future position of the stimulus correctly, and the cue is valid, or incorrectly, then the cue is invalid. Sometimes, the cue is neutral (the arrow is pointing upward) and cannot be used to infer the future position of the stimulus.

In this version of the paradigm, people can actually use the cue because it is valid most of the time (45 trials with valid cues for 15 trials with invalid cues). Therefore, the major difference between these versions of the same paradigm with spatial cuing is top‐down (controlled by individuals consciously) versus bottom‐up (grabbed involuntarily by changes in the environment, while these changes are task‐irrelevant) control of attention.

#### 2.2.6 Memory Span

Memory span refers to the ability of an individual to memorize and reproduce immediately after the presentation of a sequence of symbols in the order in which these symbols were presented (Blankenship 1938). The longer the sequence that could be reproduced correctly, the larger the memory span is, which is considered to be a proxy for working memory. A larger span provides an advantage in a variety of cognitive tasks, including reading, problem‐solving, and IQ tests. Importantly, the memory span has been reported to vary depending on the nature of the stimuli, with the span for letters being shorter than that for numbers and the span for short words being longer than that for long words (Baddeley 1994).

The participants saw a sequence of two letters of the Spanish alphabet (randomly chosen), and they had to reproduce this sequence. After two correct responses were given, the sequence of letters was incrementally increased by one letter. If a participant made a mistake two times in a row, the experiment stopped. Some participants asked an assistant to type the letters (prior to the beginning of the experiment, we verified that the participants were literate and knew the names of the letters).

### 2.3 Ethics Statement

The study was approved by the Research Ethics Committee of the Autonomous Community of Aragón (CEICA) (reference PI21/213) and will be conducted in strict accordance with the Declaration of Helsinki and Spanish regulations on personal data protection. Participation in this study was entirely voluntary, and all the subjects provided written informed consent. Confidentiality and data protection have been ensured in compliance with the General Data Protection Regulation (GDPR) (reference RAT 46/2021) (Regulation (EU) 2016/679), as well as Spanish biomedical legislation (LO 3/2018 and 14/2007).

## 3 Results

### 3.1 Psychological Measures (CRS, DASS‐21, Life Satisfaction, and UCLA Scores)

We compared the total cognitive reserve score (CRS) for men and women using independent samples *t*‐test (all pairwise tests are 2‐tailed). The assumption of normality was verified with the Shapiro–Wilks test and the assumption of equal variance of scores across both groups—with Levene's test. The CRS score was greater for women (*M *= 6.21, SD = 1.26, SE = 0.184) than for men (*M *= 5.45, SD = 1.54, SE = 0.269), and the difference between males and females was significant, *t*(78) = 2.41, *p *= 0.018, Cohen's *d *= 0.547 (based on pooled standard deviation).

**FIGURE 2:**
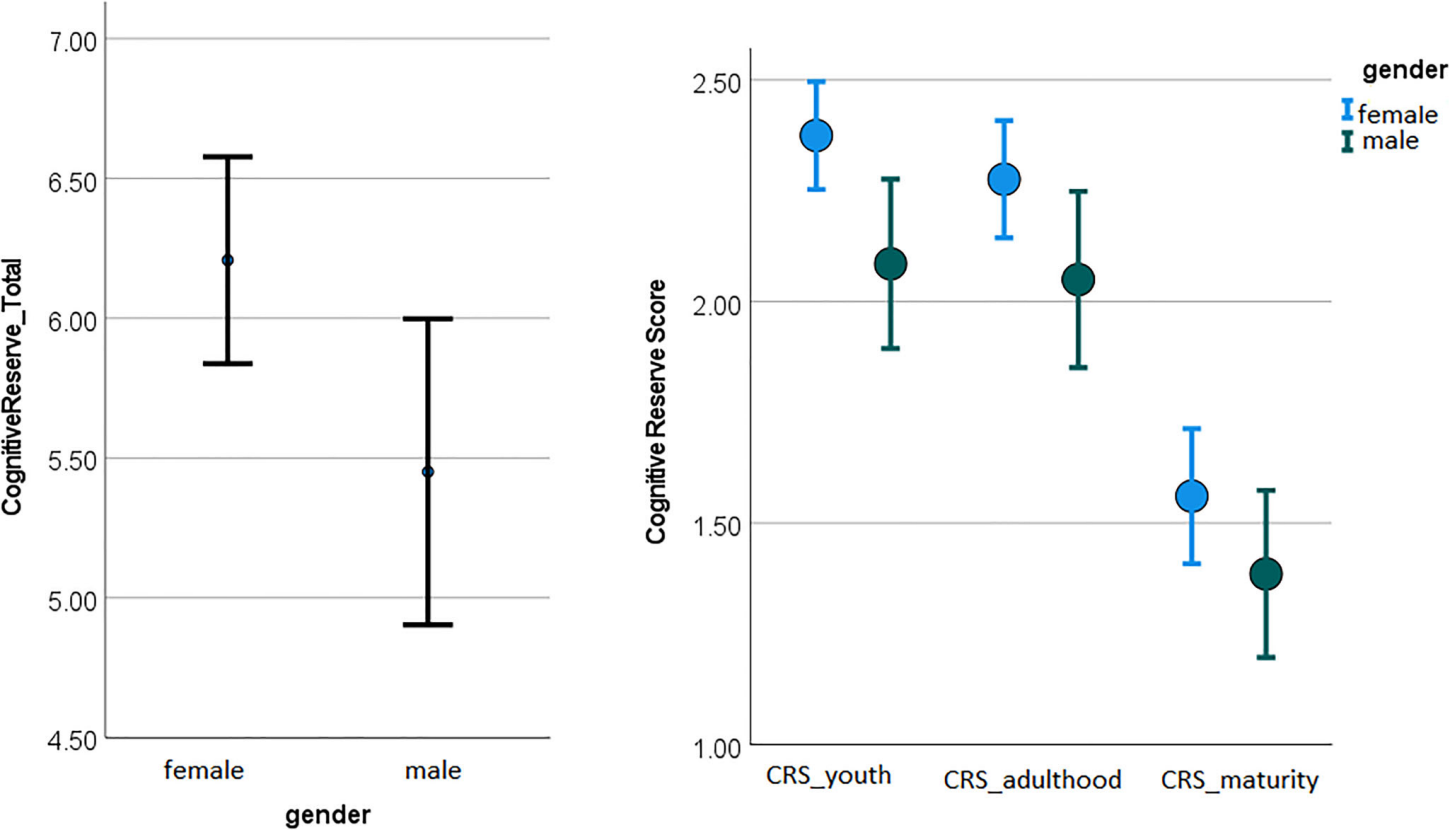
Cognitive reserve scores in male and female groups in the experimental sample. On the right, the total score on cognitive reserve (the sum of three separate scores) is displayed. On the left, we present the scores on three different measures of cognitive reserve, separately for men and women. Error bars stand for 95%CI.

Importantly, the difference in the total cognitive reserve score stems from the significant difference in the youth life stage, *t*(78) = 2.72, *p *= 0.008, Cohen's *d *= 0.617, whereas the differences in the adulthood stage, *t*(78) = 2.0, *p *= 0.049, Cohen's *d *= 0.453, and maturity life stage were not significant, *t*(78) = 1.47, *p *= 0.144, Cohen's *d *= 0.335, with the magnitude of differences between sexes decreasing with age. During the youth period, females were more likely to be engaged in activities that are reported to increase their cognitive reserve. These patterns of results are presented in Figure 2.

Importantly, we did not observe any significant correlation between the cognitive reserve and age of the participants (all *p* values are > 0.5; reported correlation coefficients are Pearson's correlation, unless otherwise specified and justified). We also performed a partial correlation between the cognitive reserve score and age, controlling for the place of residence and sex, and no significant correlations were detected, with all *p* values > 0.5. There is no association between the age of the person and the cognitive reserve score. It may suggest that the cognitive reserve does not change in the course of healthy aging, however, a longitudinal study is needed to make a strong conclusion. In addition, we correlated the cognitive reserve score with three psychological measures: life satisfaction, depression and anxiety symptoms (DASS‐21) and loneliness (UCLA) scores. We observed significant relationships neither between cognitive reserve and life satisfaction (*r *= 0.128, *p *= 0.259) nor between cognitive reserve and depression and anxiety symptoms (*r *= −0.012, *p *= 0.919). However, the correlation between loneliness and cognitive reserve was strong and highly significant (*r *= 0.341, *p *= 0.002). The cognitive reserve is greater in people who score higher on the loneliness scale.

This analysis was followed by exploratory correlations to determine if psychological well‐being is associated with cognitive reserve scores (i.e., degree of engagement in recreational activities that are known to be associated with higher cognitive reserve) differently in males and females. Splitting participants by sex, however, revealed a more intricate pattern. Although the correlations between CRS scores and DASS‐21 scores (*r *= 0.114, *p *= 0.446) and between CRS scores and UCLA scores (*r *= 0.168, *p *= 0.259) were weak and not significant for women, the correlations were strong and significant for men: greater CRS score was associated with greater loneliness, *r *= 0.555, *p* < 0.001, and psychological distress, *r *= −0.374, *p *= 0.032. We further compared the strengths of these associations. The analyses showed that the correlations between CRS scores and loneliness between male and female samples differ substantially and significantly, *z *= 1.926, *p *= .027, but the strength of correlation between CRS scores and psychological distress between male and female do not differ significantly, *z *= 1.177, *p *= 0.12.

**FIGURE 3:**
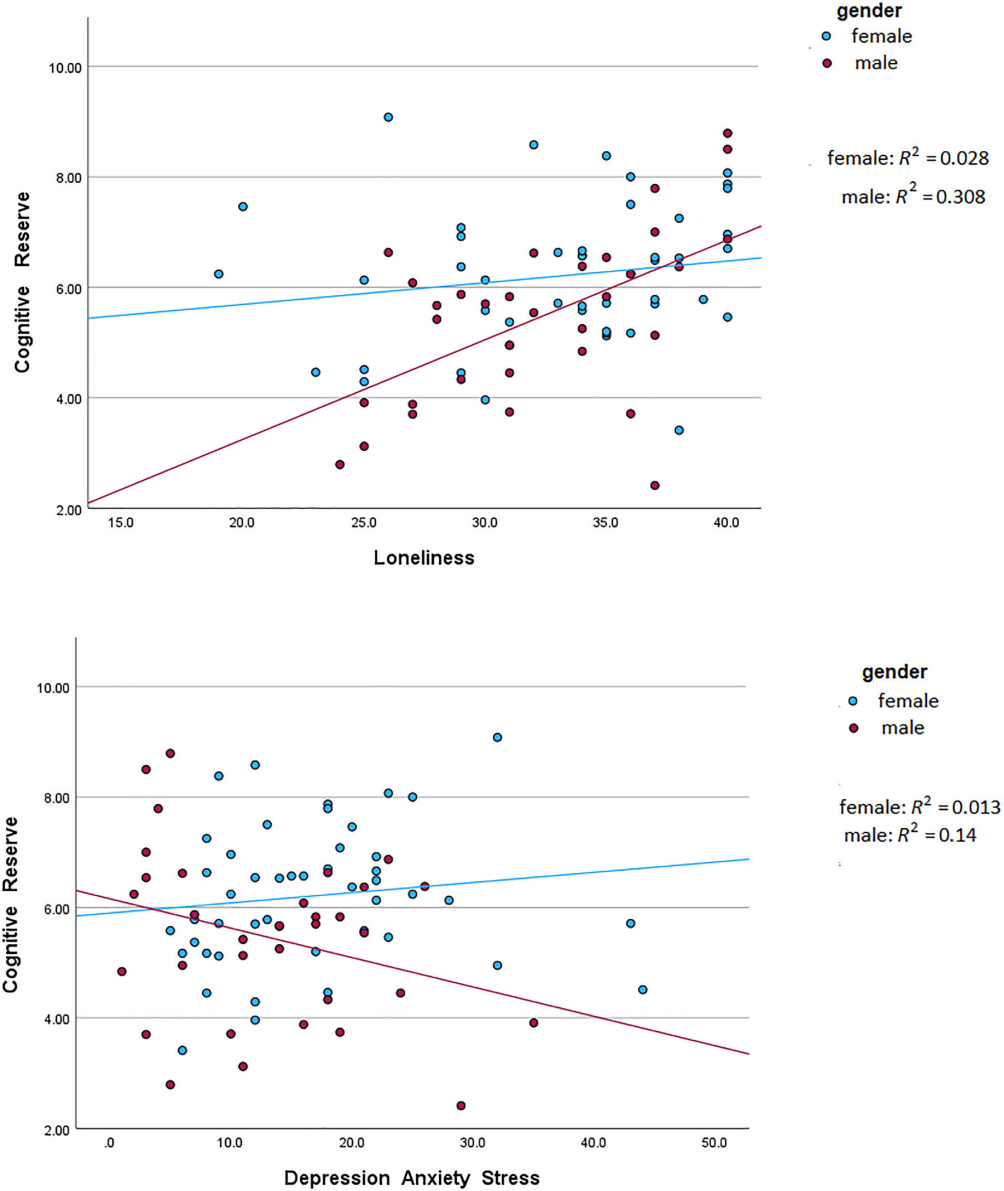
Association between loneliness and cognitive reserve (above) and between psychological stress and cognitive reserve (below) separated by sex.

These data suggest that activities associated with slower neurodegenerative processes are practiced by men whose higher loneliness score is not associated with higher scores on the DASS‐21 scale (stress and anxiety), that is, who accept their loneliness as normal and not as a reason for psychological stress. This result pattern is valid for all three measures on the CRS, as well as for the total score (see Figure 3 for associations between the total score on cognitive reserve and loneliness and between the cognitive reserve and DASS‐21 scores, which were separated into male and female subgroups in the experimental sample).

Notably, the correlation between life satisfaction and the amount of psychological stress (DASS‐21 scores) in the male population is strong and negative (higher stress is associated with lower life satisfaction), *r *= −0.482, *p *= 0.005; however, life satisfaction and the cognitive reserve are not mutually correlated, suggesting that life satisfaction and psychological stress contribute to whether males are engaged in activities that strengthen their cognitive reserve.

The conclusion that the association between psychological well‐being and the CRS scores differs between males and females, we supplemented the correlational analysis with ANCOVA, to test these effects using formal interaction models, with CRS scores as the dependent variables, biological sex as a factor, and DASS‐21 scores and UCLA scores as covariates that can modulate the relations between sex and the engagement into recreational activities associated with higher cognitive reserve (i.e., CRS scores).

The omnibus ANCOVA (full model with all interactions) revealed a significant effect of gender, F(1,72) = 4.492, *p *= 0.037, η_p_^2^ = 0.06 after controlling for subjective loneliness psychological distress. The interaction between sex and subjective loneliness was significant, F(1,72) = 5.092, *p *= 0.027, *η*_p_^2^ = 0.07, whereas the interaction between sex and psychological distress was not significant, F(1,72) = 2.28, *p *= 0.136, *η*_p_^2^ = 0.03. Three‐way interaction was not significant either. ANCOVA did not show that psychological distress was significantly associated with CRS scores after controlling for sex, F(1,72) = 1.84, *p *= 0.179, *η*_p_^2^ = 0.02, whereas significant association was observed between subjective loneliness and CRS scores, after controlling for sex, F(1,72) = 7.34, *p *= 0.008, *η*_p_^2^ = 0.09.

To account for a significant interaction, we built the model with *subjective loneliness* as a covariate and *gender* as a factor. After controlling the loneliness, the effect of gender was sill significant, F(1,76) = 7.363, *p *= 0.008, *η*_p_^2^ = 0.09. Loneliness is significantly associated with *sex*, F(1,76) = 13.89, *p* < 0.001, *η*_p_^2^ = 0.15, and significant interaction between *sex* and *loneliness*, F(1,76) = 5.73, *p *= 0.019, *η*_p_^2^ = 0.07, suggesting that the relationship between the subjective feeling of loneliness and CRS scores differs between males and females. This corroborates correlational analysis: higher subjective loneliness is related to greater engagement into recreational activities associated with higher cognitive reserve in males, but no in females. Overall, these two exploratory analyses converge and lead to the conclusion that psychological well‐being is associated with CRS scores differently in males and females, although the details of these associations and estimation of their practical significance requires further investigation.

In the second part of the analysis, we investigated the effect of cognitive reserve on performance in cognitive tests.

### 3.2 Active Attention

To analyze how cognitive reserve and top‐down directed attention are related, we used a mixed ANOVA design with accuracy (difference in correct responses on valid versus invalid cues) as a dependent variable, sex as a factor, and cognitive reserve as a covariate. Accuracy was not affected by sex, F(1,77) = 0.32, *p *= 0.57, and was not associated with cognitive reserve (total), F(1,76) = 1.83, *p *= 0.18. However, if cognitive reserve is restricted only to reporting the activities practiced in the mature life stage, the association between cognitive reserve (in maturity) and accuracy becomes significant, F(1,77) = 4.03, *p *= 0.048, *η* ^2^_p_ = 0.05, suggesting that performance in top‐down attentional regulation depends on *ongoing* mental and physical activities reported to be associated with increasing the resistance of the nervous system to withstand neurodegenerative changes. Although such activities could be practiced in earlier life stages, if the practice is stopped, the cumulative effect of practicing these activities in earlier life stages on performance in cognitive tasks gradually deteriorates with time and age.

In the next stage, we decided to analyze the effect of the cue validity. The cue is valid if the target appears where the arrow (preceding the target) is pointing. Given that the number of trials in which the cue was valid (*n* = 45) was greater than the number of trials in which the cue was invalid (*n* = 15), we compared not the number of correct responses per condition (when the cue was valid vs. when the cue was invalid) but the proportion of correct responses per condition. We did not observe any significant associations between preferential treatment of valid versus invalid cues as a function of either the cognitive reserve at maturity or the cognitive reserve total. In conclusion, we did not find evidence that valid cues improve or that invalid cues deteriorate performance in the Posner task. The improvement in the attentional task is explained by the overall improvement rather than by a preferential treatment of valid cues.

### 3.3 Passive Attention

**FIGURE 4:**
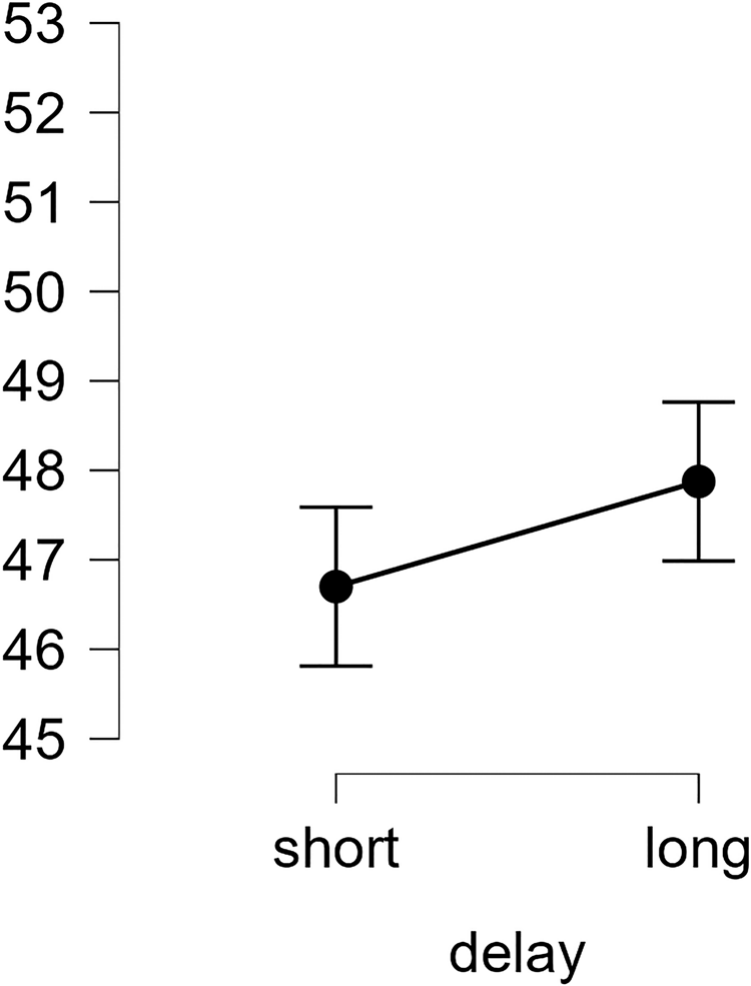
The number of correct responses as a function of the duration of the delay between the attention‐grabbing exogenous cue (non‐informative, valid in 50% of the trials) and the stimulus presentation. A longer delay allows people to disengage attention captured by non‐informative cues. Error bars stand for 95%CI.

The Posner paradigm that we used to probe passive attention allows testing how reliably people can disengage attention from non‐relevant attention‐grabbers. Attention‐grabbing cues that appear on the same side where stimuli emerge are valid, and the delay between the attention‐grabbing cue and the stimulus is either 100 ms (short delay, 60 trials) or 350 ms (long delay, 60 trials). The attention‐grabbing cues are not informative because the probability of the cue being valid is 50%. We used a repeated‐measures ANOVA to explore the number of correct responses as a function of cue validity (valid vs. invalid) and delay (short vs. long) and introduced cognitive reserve as a covariate. The analysis revealed a significant effect of delay: F(1,78) = 7.641, *p *= 0.007, *η* ^2^_p_ = 0.09; for long delays, the number of correct responses is greater than that for short delays because people have more time to disengage attention captured by non‐informative cues. Figure 4 shows that the effect is not strong (in trials with long delays, the number of correct responses is approximately 2% greater than that in trials with short delays), but a substantial effect size, *η* ^2^_p_ = 0.09, reflects that it is consistent.

Notably, the number of correct responses per delay category is modulated by cognitive reserve, F(1,78) = 5.677, *p *= 0.02, *η* ^2^_p_ = 0.07; a higher cognitive reserve increases the number of correct responses both for trials with short delays, *ρ* = 0.262, *p *= 0.019, and for long delays, *ρ* = 0.231, *p *= 0.04 (if people report to have been engaged in activities that supposedly enhance the ability of the brain to withstand neurodegenerative changes, they tend to give a larger number of correct responses, meaning that they disengage their attention from non‐informative cues more robustly). The effect of cue validity on the number of correct responses was not significant; F(1,78) = 3.622, *p *= 0.061, *η* ^2^_p_ = 0.04; the number of correct responses in trials when the cue was valid and when the cue was invalid did not differ. The interaction between cue validity and cognitive reserve was not significant either; F(1,78) = 1.925, *p *= 0.169, *η* ^2^_p_ = 0.02.

Owing to technical failure, reaction time data were not analyzed. The failure pertains to the individual behavior of some participants who, due to their inexperience with computers and old age, sometimes took disproportionately long time to respond, leading to their RT time not being recorded. We believe that RT data from such sample is not reliable due to many outlying datapoints, and accuracy data is more informative because it is less subject to age‐related response patterns that may be contaminated by age‐related experience with tasks performed under time‐pressure on computers. It is also important to note that most of our participants are coming from educational backgrounds and occupations—before retirement—were not linked to working on computers (farmers, construction workers, etc.) and the ability to handle computers (and register their responses with mouse / keyboard) was very much compromised by their life history.

### 3.4 Memory Span

We applied the Mann‒Whitney (as the assumption of normality was violated) to compare memory span between males and females. The memory span was slightly greater for males (median = 3) than for females (median = 2), but the difference was not significant (*U* = 710.5, *p *= 0.51). The correlation between memory span and cognitive reserve was negligible and insignificant (rho = 0.085, *p *= 0.454, Spearman correlation was used because the assumption of normality was violated), which does not support an association between the variables.

## 4 Discussion

Among older adults, females and males did not differ significantly in their reported engagement in activities associated with greater resistance to age‐related neurodegeneration. Although females reported greater engagement than males overall, this difference was largely confined to the younger life stage and was no longer evident in older age. The findings further indicated that, among older males, engagement in activities associated with cognitive reserve was related to psychological and social factors, including loneliness, anxiety, stress, and life satisfaction. Greater engagement was positively associated with loneliness but negatively associated with anxiety and stress. One possible interpretation is that some males experiencing loneliness may participate more frequently in recreational, social, physical, or cognitively stimulating activities. Such participation might reduce some of the adverse psychological consequences of isolation. However, the present study did not directly test whether these activities serve a compensatory function. Given the correlational nature of the findings, it is also possible that participation in these activities reduces anxiety and stress or influences perceived loneliness, rather than loneliness directly increasing engagement. Previous research has associated participation in physical and cognitively stimulating leisure activities with better psychological adjustment, well‐being, life satisfaction, and mental health after retirement in males (Şener et al. 2007; Schrempft et al. 2019). However, longitudinal or intervention studies would be required to determine the direction and mechanisms of these relationships and whether the indeed may help mitigate some of the negative consequences of social isolation and subjective loneliness in older males.

Other explanations might be related to solitary cognitive activities beyond those included into CRS measurements (Song et al. 2022). However, in the female sample, such associations were not found. We hypothesize that internal motivation is more robust in females and that they do what they feel doing, irrespective of their environmental factors and life circumstances (e.g., if they like exercising or socializing, they are more likely to continue to be engaged in these activities when life circumstances change). Sex differences might also pertain to differences in or personality traits or in social network structure of males and females, as suggested by Oh et al. (2021). Hwang et al. (2018) showed that social activity may not have an impact on the decline of cognitive function for men, although women are negatively affected by social isolation or subjective feeling of loneliness. Males might redirect time resources freed from lower participation in social gatherings into other recreational activities, some of which are associated with building cognitive reserve. Potentially, education might also play a role, however our participants had maximum secondary school education, and most attended only primary educational institutions (Song et al. 2022). We do not have variability in educational level in our sample.

Several additional explanations should be considered. Reported engagement into activities may reflect gender roles typical of their generation rather than general differences between females and males, hence the observed sex‐specific patterns may reflect cohort effects. In addition, males and females may differ in how they interpret or report loneliness, anxiety, stress, and leisure participation. Importantly, we cannot exclude the reporting bias introduced by the sampling method. The volunteers for this study were capable of living independently. Individuals experiencing more severe social isolation, cognitive impairment, poor health, or restricted mobility may consequently have been less likely to participate. This potential sampling bias might limit the generalizability of the observed associations.

Despite acknowledged limitations, the overall result pattern is in line with earlier findings reporting that, on average, females resist age‐related neurodegeneration more efficiently (Chen et al. 2017; Wang et al. 2020) because they are less susceptible to external factors pertaining to subjective psychological well‐being. The origin of this difference is more likely to be social (sex‐related influence) rather than biological (sex‐related influence), although this hypothesis still needs to be tested.

The data also showed that greater engagement in activities measured by the CRS during the mature life stage was associated with more efficient performance on measures of attentional disengagement and the use of predictive cues. In contrast, reported engagement during the younger life stage was not significantly associated with current cognitive performance. This pattern suggests that activity engagement closer to the time of cognitive assessment may be more closely related to current performance than retrospectively reported engagement earlier in life. However, these findings do not demonstrate that discontinuing an activity causes cognitive benefits to disappear, nor do they establish that sustained engagement protects against cognitive decline. The data were cross‐sectional, and activity engagement at earlier life stages was assessed retrospectively. It is therefore possible that individuals with better preserved cognitive functioning are more able or more inclined to continue participating in physical, social, and cognitively stimulating activities. Nevertheless, this conclusion is in line with detraining studies showing that cognitive benefits after exercise may decline after training cessation, while longitudinal studies show that continued participation in physical, social, cultural, and cognitive activities in later life is associated with better cognitive performance and slower cognitive decline (Timmons et al. 2020). Ferreira et al. (2024), based on systematic review of detraining studies, concluded that detraining periods of 16 weeks can negatively affect cognitive (especially executive) functions. We suggest that only continuous engagement in physical activities, social life or cognitive and creative tasks by older adults is associated with better cognitive performance, which echoes the concluding emphasis of Timmons et al. (2020) on the importance of long‐term exercise maintenance in older adults.

However, whether performance in cognitive tasks can be improved by such activities if an older individual never (or irregularly/infrequently) practiced them before needs to be explored. This question cannot be addressed in our sample because we either have individuals who practiced activities associated with better resistance to neurodegeneration in younger life stages and stopped or reduced the frequency of these activities at maturity or who did not practice these activities in the youth and did not start practicing them later in life.

In summary, the data seem to indicate that sustaining cognitive functionality is associated with engagement in activities measured by the CRS, which, in turn, is also related to participants’ psychological well‐being and sex (Hsieh et al. 2024; Yao et al. 2024).

The validity of the conclusions is limited by several considerations. The data is cross‐sectional, and the only longitudinal information is a self‐report of how much the participants practiced activities associated with decelerated neurodegeneration at different life stages. We acknowledge that personal report is not the most reliable measure, although it does provide some insight into whether continued practice throughout life is important for cognitive performance or whether practicing at a younger age will have a long‐lasting effect and be a substitute for life‐long exercise. None of our participants resided in assisted living facilities or had caretakers who supported their day‐to‐day life, yet many people with mild cognitive impairments with high cognitive reserve are able to sustain independent living. Finally, we do not have biological data from participants (MRI scans or biological data samples for objective assessment of structural and functional neurodegenerative changes and the metabolic state of the central nervous system) or assessment of their cognitive health. Neither do we measure cognitive reserve directly; we use the CRS scale, which reflects the frequency with which people practice activities that are correlated with stronger resistance of the neural system to neurodegeneration. Despite the high test‒retest consistency of the scale (León et al. 2014, 2017), its customary use in research on healthy aging, and multiple evidence that practicing such activities is indeed associated with greater cognitive reserve (Wilson et al. 2003; Scarmeas et al. 2003), this is not a direct measure of the brain's ability to resist neurodegeneration. The causal relationships between the likelihood of practicing such activities and the biological (neuroanatomical and neurophysiological) properties of the neural system to withstand age‐related changes are not clear. It is possible that older adults whose neural system is more resilient to degeneration continue practicing social, physical and cognitive activities. The CRS score is often used as a reliable proxy measure of cognitive reserve, and we follow this trend in our study but without postulating causal relationships between the CRS items and neurobiological predispositions or age‐related changes. We say that people have high cognitive reserve if they practice certain activities but not because they practice these activities. Given these limitations, empirical studies would be useful for verification and generalization of these conclusions, as well as to empirically test further hypotheses that can be generated on the basis of these conclusions.

Future studies will have to include a wider range of cognitive performance tasks (beyond testing the operational memory span and attentional resources and ideally including higher‐level cognitive tasks probing decision‐making and information processing). Moreover, more direct measures of cognitive reserve are needed to explore the anatomical and physiological correlates of resistance to age‐related neurodegenerative processes in healthy aging (e.g., analysis of blood samples, anatomical and physiological PET/MRI images, electrophysiology of cognitive processing, and changes in body functioning before vs. after physical stress). Furthermore, we must include environmental factors, e.g., air quality, which are known to directly affect cognitive reserve (López‐Granero et al. 2024; Hsieh et al. 2021), sometimes without affecting proxy measures such as the frequency of engagement in physical, social and cognitive activities that are associated with better cognitive reserve. Finally, including a wider range of variables requires increasing the sample size of the older people five‐to‐eight times (*n* = 400–600 participants). This larger‐scale study will allow the design of intervention programs aimed at boosting the cognitive reserve by targeting lifestyle and psychological well‐being while considering anatomical/physiological idiosyncratic predispositions and environmental factors characteristic of an individual's place of residence.

## Author Contributions

**Mikhail Ordin**: investigation, writing – original draft, formal analysis. **Angel Barrasa**: resources, writing – review and editing. **Caridad López‐Granero**: conceptualization, investigation, funding acquisition, data curation, supervision. **Leona Polyanskaya**: conceptualization, investigation, funding acquisition, data curation, writing – review and editing.

## Funding

This work was funded by the Spanish Government (Ministry of Science and Innovation; MCIN/AEI/10.13039/501100011033) (Grant RYC2022‐037025‐I (LP) and PID2020‐113812RA‐C33 (C.L‐G.)).

## Ethics Statement

The study was approved by the Research Ethics Committee of the Autonomous Community of Aragón (CEICA) (reference PI21/213) and will be conducted in strict accordance with the Declaration of Helsinki and Spanish regulations on personal data protection. Participation in this study was entirely voluntary, and all the subjects provided written informed consent. Confidentiality and data protection have been ensured in compliance with the General Data Protection Regulation (GDPR) (reference RAT 46/2021) (Regulation (EU) 2016/679), as well as Spanish biomedical legislation (LO 3/2018 and 14/2007).

## Conflicts of Interest

The authors declare no conflicts of interest.

## Data Availability

The data that support the findings of this study are available from the corresponding author upon reasonable request, and restrictions will apply with respect to further sharing the data. Data reuse will be possible considering that researchers follow the principle of the minimal dataset required to test new hypotheses, even if the full dataset is shared by the corresponding author. Restrictions on resharing might apply due to the sensitive nature of the data.
