# Corrections to “Cognitive Reserve and Psychological Well‐Being in Aging Population of Community‐Dwellers in Spain”

**DOI:** 10.1002/brb3.71860

**Published:** 2026-10-18

Ordin, M., Barrasa, A., López‐Granero, C., & Polyanskaya, L. (2026). Cognitive Reserve and Psychological Well‐Being in Aging Population of Community‐Dwellers in Spain. *Brain and Behavior* 16, no. 9: e71714. https://doi.org/10.1002/brb3.71714

The affiliation for author Caridad López‐Granero was incorrect. The correct affiliation is 4: Department of Psychology and Sociology, Universidad de Zaragoza, Teruel, Spain (previously listed as 3).

Additionally, an inadvertently omitted funding source has been added to the Funding section. The corrected funding statement should read:

“This work was funded by the Spanish Government (Ministry of Science and Innovation; MCIN/AEI/10.13039/501100011033) (PID2020‐113812RA‐C33 [C.L‐G.]), Grant RYC2022‐037025‐I (L.P.), and Gobierno de Aragón (Grant PROY_S12_24 [C.L‐G]).”

We apologize for these errors.

